# Host-Guest Complexes of Flavanone and 4′-Chloroflavanone with Naturals and Modified Cyclodextrin: A Calorimetric and Spectroscopy Investigations

**DOI:** 10.3390/molecules29133123

**Published:** 2024-06-30

**Authors:** Artur Stepniak, Marta Biernacka, Magdalena Malecka, Bartlomiej Palecz

**Affiliations:** Unit of Biophysical Chemistry, Department of Physical Chemistry, Faculty of Chemistry, University of Lodz, Pomorska 163/165, 90-230 Lodz, Polandmagdalena.malecka@chemia.uni.lodz.pl (M.M.); bartlomiej.palecz@chemia.uni.lodz.pl (B.P.)

**Keywords:** solubility enhancement, cyclodextrin, inclusion complex, Higuchi-Connors method, isothermal titration calorimetry, DSC, flavanone

## Abstract

The aim of the research was to investigate and compare the interaction between flavanones (flavanone, 4-chloro-flavanone) with potential anticancer activity and selected cyclodextrins. Measurements were made using calorimetric (ITC, DSC) and spectrophotometric (UV-Vis spectroscopy, FT-IR, ^1^H NMR) methods. The increase in the solubility in aqueous medium caused by the complexation process was determined by the Higuchi-Connors method. As a result of the study, the stoichiometry and thermodynamics of the complexation reaction were determined. The formation of stable inclusion complexes at a 1:1 M ratio between flavanone and 4-chloroflavanone and the cyclodextrins selected for research was also confirmed.

## 1. Introduction

A significant problem for the modern pharmaceutical industry is the limited solubility of some APIs (active pharmaceutical ingredients) in an aqueous environment. Solubility plays a key role in the effectiveness of a drug. Low solubility is usually associated with limited absorption in the body, which may lead to suboptimal delivery and absorption of the drug, resulting in its ineffectiveness and many side effects. This also raises other problems. Among others, they are related to metabolism or permeability, interactions with other drugs, or the need for sustained drug release.

Developing an appropriate recipe for a new drug while maintaining adequate effectiveness can be a challenge. There are many methods to increase API solubility, including salt formation, size reduction, prodrug formation, and many others [1,2,3,4,5,6]. One of them is complexation inside other particles, e.g., cyclodextrins.

Cyclodextrins are cyclic polysaccharides that are one of the most studied macrocycles in macromolecular chemistry. The cyclodextrin molecule has a ring structure made up of glucose units. Natural α-, β-, and γ-cyclodextrins differ from each other in the number of glucose units. The α-, β-, and γ- cylodextrins correspond to six, seven, and eight glucose units, respectively [7]. Cyclodextrins have a characteristic cavity in their structure, which allows molecules of apolar compounds to be located within them [8,9]. The formation of connections is based on the formation of weak intermolecular interactions, mainly hydrophobic interactions, van der Waals, and hydrogen bonds [10]. The formation of a cyclodextrin complex with a ligand molecule can change its physicochemical properties, such as its thermodynamic stability or solubility. Compared to other macromolecules, they are distinguished by a lack of toxicity [11,12] and the possibility of universal application in many fields of industry. The formation of inclusion complexes with hydrophobic compounds gives the opportunity to use cyclodextrins as carriers in medicine, cosmetics, and agriculture [13,14,15,16]. In addition to increasing solubility, complexation also protects the API molecule against external factors (temperature, humidity) and also allows the formulation of drugs in solid form when necessary, and also acts as a natural preservative and emulsion stabilizer; can also control the release time of the drug and minimize side effects [17,18,19,20]. Cyclodextrins can mask the unpleasant taste of drugs by encapsulating them in their cavity and preventing them from interacting with taste receptors in the mouth [21].

In the presented research, the role of the ligand enclosed in the interior of cyclodextrins was played by flavanone and 4′-chloroflavanone.

**Figure 1:**
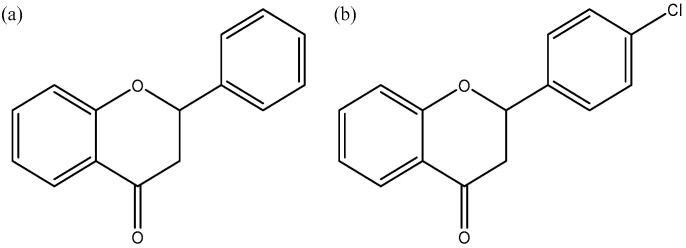
Structure of selected ligands: (**a**) flavanone (FL0), (**b**) 4′-chloroflavanone (FL2).

Flavanones are compounds of the group of flavonoids. Their spatial structure is based on a 4-chromanone ring. Naturally occurring flavanones are, among others, naringenin or hesperetin. Flavanones naturally occur as secondary metabolites produced by plants. Their main source is citrus fruits, for example, grapefruit and oranges. These compounds have antioxidant, anti-inflammatory, anticancer, and cardioprotective properties. Studies show that they can regulate the concentration of the LDL cholesterol fraction in the body [22]. Two compounds were selected for the study: the basic unsubstituted flavanone molecule and its derivative, chlorine substituted in the 4′ position (Figure 1). The selected compounds show potential use in medicine. 4′-chloroflavanone inhibits the proliferation of breast cancer cells of both MCF-7 and MDA-MB231 lines and has a toxic effect against many human tumor cell lines: MCF-7, MDA-MB231, as well as LNCaP and PC3 (prostate cancer cell lines) and also HepG2 (liver cancer cell line), SK-N-MC (neuroblastoma cell line), K-562 (leukemia cell line), and KB (nasopharyngeal epidermal cancer cell line) [23,24]. 

Investigating the interactions between flavanones and cyclodextrins seems interesting and has a lot of motivation. First, the possibility of creating a complex may be a carrier of these compounds in further potential use in medicine. However, research on complexes between selected compounds, which are some basic molecules of the flavanone group, may be of key importance as a starting point for the analysis of complexes with flavanone with other types of substituents. Research will allow the evaluation of the influence of the substituent in the 4 position on the ability to form inclusion complexes with cyclodextrins.

The research presented aimed to investigate the interaction between selected cyclodextrins (α-cyclodextrin, β-cyclodextrin, and hydroxypropyl-β-cyclodextrin) and selected flavanones: flavanone (FL0) and 4′-chloroflavanone (FL2). Calorimetric titrations (ITC) were performed to determine the thermodynamic parameters of the obtained complexes. Functions describing the formation of the complex were determined, such as changes in entropy, enthalpy, and Gibbs free energy as well as the stability constants of the complexes. Due to the extremely low solubility of 4′-chloroflavanone in water, all calorimetric titrations were performed in DMSO. Dimethyl sulfoxide is a universal solvent, often used as a replacement for water-insoluble substances. It is characterized by low toxicity [25]. DMSO is also used in medicine as a carrier of some drugs [26] and as a cryoprotectant [27]. To determine the increase in the solubility of flavanones in water, phase equilibrium studies were carried out by the Higuchi-Connors method using ultraviolet (UV) spectroscopy. DSC analysis was also performed to determine the effect of complexation on the thermal stability of the compounds.

## 2. Results and Discussion

### 2.1. Isothermal Titration Calorimetry (ITC)

**Figure 2:**
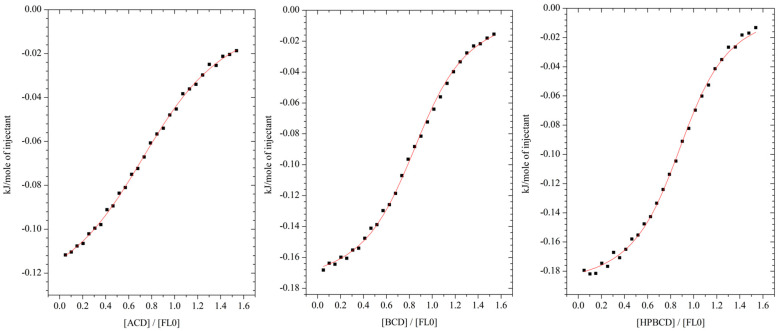
Energy effects of the direct interaction of flavanone (FL0) with cyclodextrins in DMSO.

The thermal effects describing the direct interaction of flavanone (FL0) with α-CD, β-CD, and HP-β-CD (Mw approximately 1380) in dimethylsulfoxide (DMSO) as a function of the solution are shown in Figure 2.

For the mathematical description of the thermograms, the model of one active site was used. Based on this model, the stoichiometry of the inclusion complex (n), the formation constant of the flavanone (FL)-cyclodextrin complex (K), the molar enthalpy (ΔH), and the entropy (ΔS) of the complexation process were determined.

The Gibbs standard free energy of binding, ΔG, was calculated using the basic thermodynamic relationships:

∆G=∆H−T∆S=−RTln(K)

where: T—temperature, R—gas constant, K—constant of complex.

The values of the thermodynamic parameters that describe the interaction of flavanone (FL0) with α-, β-, and HP-β-cyclodextrin in dimethylsulfoxide are presented in Table 1. The stoichiometric ratio (n), denoting the number of flavanone molecules (FL0) per cyclodextrin macromolecule, was close to one in all FL0-CD connections.

**Figure 3:**
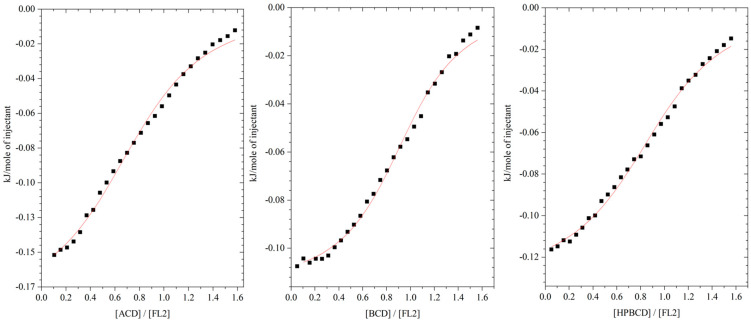
Energy effects of the direct interaction of 4′-chloroflavanone (FL2) with cyclodextrins in DMSO.

The obtained values of (n) (Table 1) suggest the formation of flavanone-cyclodextrin complexes with a stoichiometric ratio of 1:1. All analyzed processes are exothermic (∆H < 0) and spontaneous (∆G < 0) (Figure 2 and Figure 3). The obtained results showed that the complex formation of α-, β-, and hydroxypropyl-β-cyclodextrin with both analyzed flavanones is driven by entropy. This indicated that the difference in cavity size is not reflected in the different driving forces of complex formation, which resulted in the same stoichiometry of all complexes obtained. The values of the formation constants allow us to conclude a stronger interaction between the ligand and β-cyclodextrin than the α-cyclodextrin. The stability constant of the complex with hydroxypropyl-β-cyclodextrin has a lower value compared to the unsubstituted β-cyclodextrin molecule. This is most likely due to the steric hindrance caused by hydroxypropyl groups, which hinders the complex formation process [28,29].

The values of the thermodynamic parameters that describe the formed complexes were determined based on the model of an active site, the same way as in the case of flavanone (FL0) (Table 2).

The determined stoichiometric ratios of ligand-receptor in the analysis connection systems indicate the formation of a 1:1 complex. The molar enthalpy values (ΔH < 0) of the reactions examined indicate the exothermic nature of the complexation processes and the free molar enthalpies (ΔG < 0) of their spontaneous nature.

### 2.2. Differential Scanning Calorimetry (DSC)

Based on results from ITC and UV-Vis studies, differential scanning calorimetry measurements were performed for the complexes of both flavonoids for a 1:1 molar ratio.

**Figure 4:**
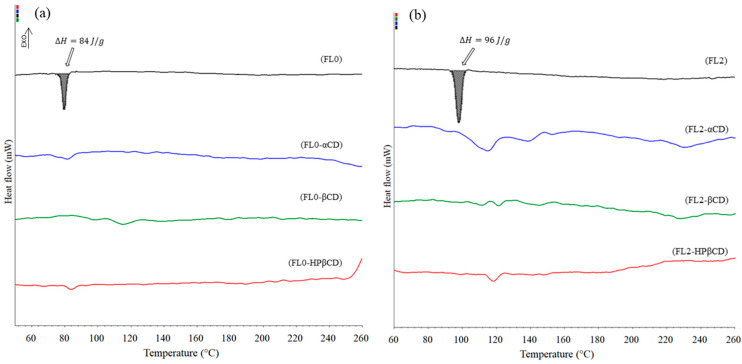
Thermograms of pure flavanones and their complexes: (**a**) flavanone (FL0); (**b**) 4′-chloroflavanone (FL2) (thermograms of pure cyclodextrins in Supplementary Materials).

The pure flavanone thermogram presented in Figure 4a (FL0 curve) shows an endothermic peak at a temperature of 77–78 °C, corresponding to the melting point of this compound (Table 3). On thermograms of the flavanone (FL0) complexes with α-, β- and hydroxypropyl-β-cyclodextrin, a clear reduction and widening of the effects related to the melting of the compound can be observed. There is also a noticeable shift of the peak towards higher temperatures for the βCD complex [30].

Similar effects were observed for 4′-chloroflavanone (Figure 4b). The melting points of the pure compound correspond to the data from the literature (Table 3). Shifts in melting points towards higher values by about 20–40 degrees were observed in the case of the complexes. A reduction in the area of endothermic peaks was also observed relative to that of the pure, uncomplexed compound. 

The DSC thermogram of the flavanone complex with cyclodextrin significantly reduces the intensity of the peaks, and their shift toward higher temperatures confirms the formation of complexes in the solid form between the flavanones and cyclodextrins [31].

**Table 3:** Melting points of flavanone and 4′chloroflavanone.

Melting Temperatures of Flavanones		
	Experimental Values	Literature Values
FL 0	77–78 °C	75–77 °C [32], 76–77 °C [33]
FL 2	96–97 °C	95–97 °C [32], 94–96 °C [34]

### 2.3. UV-Vis Spectroscopy (Phase Solubility Study)

**Figure 5:**
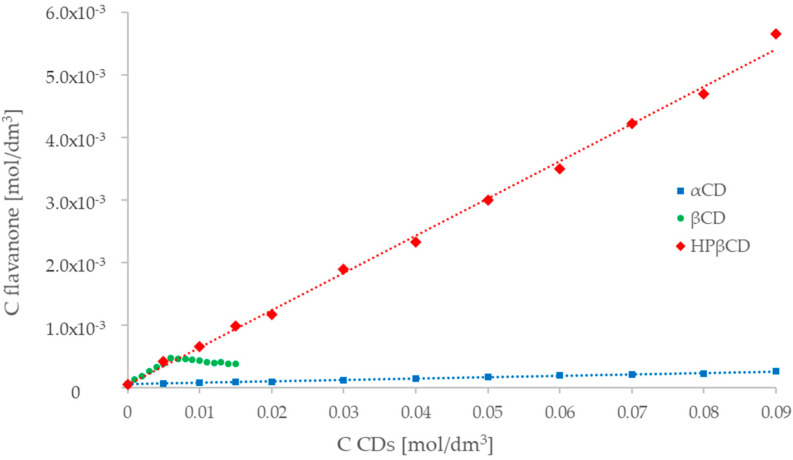
Dependence of the flavanone concentration (FL0) on the concentration of selected cyclodextrins (**▪** α-cyclodextrin, ● β-cyclodextrin, 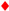 HP-βcyclodextrin).

Phase equilibrium studies for flavanone (FL0) with α-cyclodextrin as a solubilizer allowed one to achieve an approximately 5-fold increase in the solubility of flavanone in water. The relationship obtained between the concentration of flavanone and the concentration of cyclodextrin was linear (A_L_ type curve [35]) (Figure 5). For β-cyclodextrin, an 8-fold increase in the solubility of flavanone was obtained. However, the relationship C_βCD_ = f(C_FL0_) (Figure 5) was linear only in the macrocycle concentration range of up to 6 mM, and the higher concentration of β-cyclodextrin did not cause a further increase in the solubility of flavanone (FL0).

The presence of such a plateau for A_L_ curves is observed when the complexing compound (cyclodextrin) is not freely soluble in water. The complexed compound (FL0) is saturated, and maximum solubility is reached [34]. Due to the relatively low water solubility of β-cyclodextrin, it can be concluded that this is the case for the FL0–βCD combination.

The results obtained for the flavanone complex (FL0) with the highly soluble hydroxypropyl derivative of β-cyclodextrin confirm this assumption. In the case of this cyclodextrin, linear relationships were obtained in the full range of macrocycle concentration (up to 90 mM) (Figure 5). It allowed a 100-fold increase in the solubility of the ligand in water [36].

For all cyclodextrins, a linear increase in the solubility of flavanone (FL0) can be observed, and the solubility diagrams indicate the type of A_L_ curve, which indicates the formation of complexes with a stoichiometric ratio of 1:1 like naringin [37].

The equations of the lines obtained allowed us to determine the stability constants of the complexes using the Higuchi-Connors Equation (1), where slope—tg α of the ligand concentration versus the macrocycle concentration curve.; S_0_—the intrinsic solubility of the ligand in water without the addition of cyclodextrin [35].

(1) $$K_{1:1}=\frac{Slope}{S_{0}\left(1-Slope\right)}$$

For 1:1 complexes, the complexation efficiency (CE) can be calculated from the slope of the phase-solubility diagram in Equation (2)

(2) $$CE{=S_{0}\cdot K}_{1:1}=\frac{Slope}{\left(1-Slope\right)}$$

The values of the stability constants, the complexation efficiency of the complexes, and the increase in the water solubility of flavanone caused by inclusion in the cyclodextrin molecule are presented in Table 4.

Comparing the results obtained for natural cyclodextrins, it can be seen that a larger beta-cyclodextrin molecule (despite the use of a lower concentration) is able to induce a twice greater increase in solubility compared to α-cyclodextrin. The formation constants (Table 4) also indicate the formation of a complex with negligible stability between the α form and the flavanone (FL0), compared to β-cyclodextrin, for which the constant value is K > 1000 dm^3^mol^−1^, which proves the formation of a stable complex. The use of the β cyclodextrin derivative increases the solubilization of flavanone while maintaining the stability of the complex.

**Figure 6:**
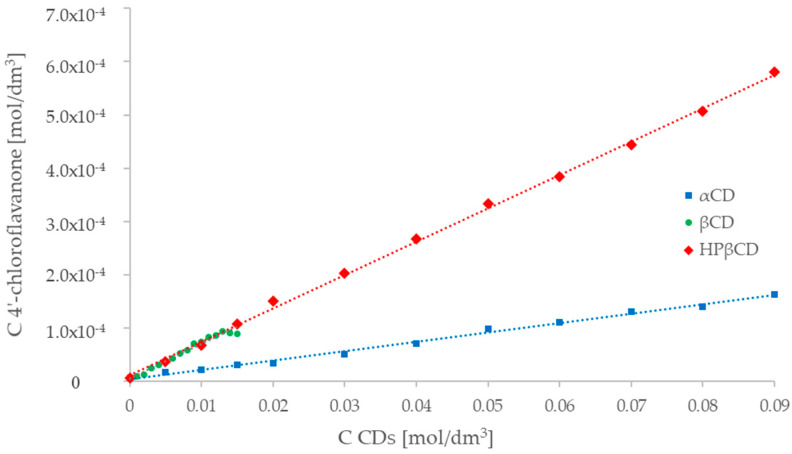
Dependence of the concentration of 4′-chloroflavanone (FL2) on the concentration of selected cyclodextrins (**▪** α-cyclodextrin, ● β-cyclodextrin, 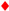 HP-βcyclodextrin).

In the case of 4′-chloroflavanone, phase solubility studies for the three cyclodextrins indicate the formation of complexes with a stoichiometric ratio of 1: 1 (linear relationship—type of A_L_—curve) (Figure 6). The maximum increase in the solubility of the ligand (FL2) in water for α-cyclodextrin was 27 (Table 5). β-cyclodextrin as a solubilizer, similar to that discussed above, allowed archiving a linear relationship with a plateau, but at a macrocycle concentration of 13 mM (Figure 6). The use of β-cyclodextrin allowed a 17-fold increase in the concentration of 4′-chloroflavanone in water. Hydroxypropyl-β-cyclodextrin caused a 140-fold increase in the solubility of the ligand (FL2) in water, and the dependence obtained takes the typical shape of the A_L_ curves in the entire range of concentration of cyclodextrin (Figure 6). 

The determined values of the formation constants are presented in Table 5. The stability constant of the α-cyclodextrin inclusion complex with 4′-chloroflavanone (FL2) has a greater value than the flavanone complex (FL0). The stability constants for β-cyclodextrin and HP-β-cyclodextrin assume similar values.

When comparing the results obtained in the range of 0–15 mM (Figure 5 and Figure 6) for natural cyclodextrins, it can be seen that β-cyclodextrin causes a greater increase in solubility than the α form. This shows a better spatial fit between the flavanone molecules and β-cyclodextrin. This is confirmed by the determined stability constants (Table 6), which for α-cyclodextrin have lower values [36].

It was observed that the addition of β-cyclodextrin plateaued the solubility curves, which limited the increase in ligand concentration. This phenomenon is observed in the case, of using a solubilizer (in this case β-cyclodextrin) with relatively low solubility [28]. The use of highly soluble hydroxypropyl-β-cyclodextrin did not limit the increase in flavanone solubility. Furthermore, it was observed that the concentration of β-cyclodextrin at which flattening occurs is probably related to the solubility of the pure compound in water. The greater the solubility of the flavanone, the more flattening occurs at a lower concentration of cyclodextrin.

In the case of 4′-chloroflavanone (FL2), α-cyclodextrin causes a greater increase in solubility in relation to FL0 flavanone, moreover, it has almost 8 times higher values of the stability constant compared to FL0. This is due to the presence of the chlorine atom in the para position in the C-ring of the flavanone. The presence of this substituent most likely stabilizes the complex by creating hydrogen bonds between the chlorine atom and the hydrogen atoms in the cyclodextrin molecule.

When comparing the obtained values of the increase in solubility of both tested flavanones for β-cyclodextrin and hydroxypropyl-β-cyclodextrin in the range of 0–15 mM, an almost identical course of the curves can be observed. Differences in concentration increase are only due to the presence of a plateau for the native β form. The use of hydroxypropyl-β-cyclodextrin makes it possible to be carried out in the concentration range of 0–90 mM, allowing an approximately 100-fold increase in the solubility of the ligand in water. The stability constants obtained for β-cyclodextrin, and the substituted derivative assume similar values.

### 2.4. FT-IR Spectroscopy

The spectra of pure flavanones are characterized by typical bands corresponding to data from the literature [32,38]. The values obtained for both flavanones are presented below:

flavanone: 1690.2 cm^−1^; 1605.8 cm^−1^; 1462.4 cm^−1^; 1303.7 cm^−1^; 766.7 cm^−1^; 487.8 cm^−1^ [38]
4′-chloroflavanone: 1693.9 cm^−1^; 1599.2 cm^−1^; 1461.9 cm^−1^; 1300.2 cm^−1^; 1121.1 cm^−1^; 905.9 cm^−1^; 847.5 cm^−1^; 773.1 cm^−1^ [32]

**Figure 7:**
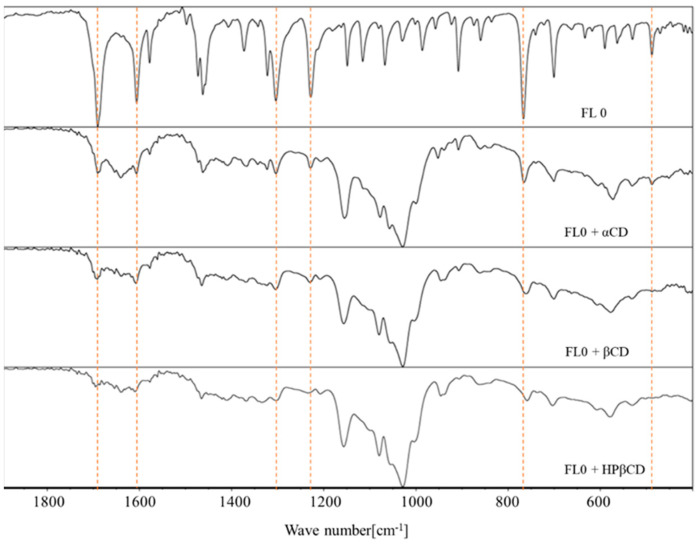
The FTIR spectrum of flavanone (FL0) and its complexes.

**Figure 8:**
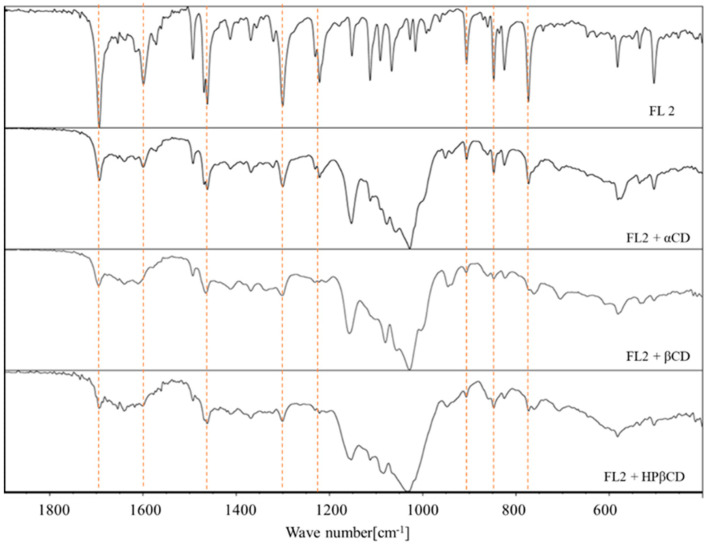
The FTIR spectrum of 4′-chloroflavanone (FL2) and its complexes.

In the spectra of the complexes (Figure 7 and Figure 8), a decrease in the characteristic bands of the flavanones was observed. Changes in the FT-IR spectra were also observed by Kim [39] and Qiu et al. [40]. The authors observed displacement, reduction, and disappearance of the peaks in the complex of cyclodextrin. This indicates the inclusion of flavanone molecules in the hydrophobic interiors of the macrocycles. The characteristic bands of flavanones are more visible in complexes with α-cyclodextrin compared to those of complexes formed by beta and hydroxypropyl-β-cyclodextrin (FTIR spectrum of pure cyclodextrins in Supplementary Materials). This may indicate that fewer ligand molecules stably bind to α-cyclodextrin in comparison to its larger derivatives and consequently confirm the formation of more stable complexes using the β form, in which more flavanone molecules have been incorporated.

### 2.5. ^1^H NMR Spectroscopy

In this work, we confirmed the formation of interactions in the complexes and examined them using the ^1^H NMR spectroscopy.

**Figure 9:**
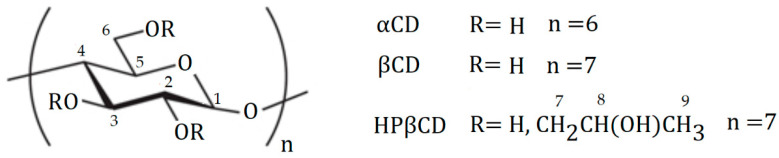
Proton numbering in the structure of cyclodextrins.

The ^1^H NMR experiment allows us to observe differences in proton shifts between free molecules and complexes. Shifts in protons for cyclodextrins allow us to assess whether the complex is forming and to conclude about the location of the molecule inside the cavity of the cyclodextrin molecule (Figure 9). Changes in chemical shifts for protons H3 and H5 (inside the cavity) take place in the presence of aromatic rings of the ligand molecule due to the anisotropic effect of the aromatic ring [41]. It was observed that when δ H3 > δ H5, partial inclusion of the guests into the cyclodextrin cavity takes place, and when δ H3 < δ H5, the molecule includes completely [42].

The hydrogen chemical shift (δ) and chemical shift differences (Δδ) of CDs and their complexes are summarized in Table 7 (^1^H NMR spectra in Supplementary Materials).

The presence of chemical shift differences indicates the formation of interactions between cyclodextrins and flavanones. Similar results could be found in literature as evidence of formation complexes [43]. The chemical shift of the H3 protons of cyclodextrin changes the most, indicating that mainly these protons contribute to the formation of complexes. In all cases, we observed a greater shift for H3 than for H5, which indicates partial inclusion of the complexes.

Moreover, greater differences can be observed for βCD complexes than for αCD complexes. This indicates a better fit of flavanone molecules to the interior of βCD. The trend of shift differences confirms our conclusions from previous methods (ITC, UV-Vis spectroscopy) and indicates the formation of the strongest interactions in the following order: βCD > HPβCD > αCD.

## 3. Materials and Methods

### 3.1. Materials

The materials used in this study were as follows: α-cyclodextrin (purity > 98%), β-cyclodextrin (>98%), HP-β-cyclodextrin (99%) (TCI), flavanone, and 4′-chloroflavanone. The substances used for the tests were dried in a vacuum dryer, cyclodextrins at 100 °C flavanone, and 4′-chloroflavanone at 50 °C. The water used for the spectrophotometric measurements of UV-Vis spectroscopy was triple distilled and degassed.

Cyclodextrin complexes in the solid state (for DSC and FTIR studies) were prepared by the co-evaporation method. Ligands (flavanone and 4′-chloroflavanone) were dissolved in ethanol, while cyclodextrins were dissolved in triple distilled water. The ethanolic solution of flavanones was gradually added dropwise into the aqueous cyclodextrin solutions. The solutions prepared this way were heated to 40 °C and mixed on a magnetic stirrer for about 48 h. The solvent was completely evaporated from the complex solution (72 h at 70 °C). The ethanol content in the solid-state complex was determined using the TG-DSC 111 (Setaram, Caluire-et-Cuire, France) thermobalance. We did not observe mass loss within the temperature range of 70 to 85 °C, indicating the absence of ethanol in the complex samples tested.

### 3.2. Isothermal Titration Calorimetry (ITC)

All isothermal calorimetric titration measurements were performed at 25 °C using a Microcal VP-ITC (Malvern, Worcestershire, UK) microcalorimeter. Because 4′-chloroflavanone has very low solubility in water, for measurements for both flavanones, DMSO was used as a solvent. The ligand solution, with a concentration of (5.23 mM FL0 and 5.23 mM FL2), was placed in the measuring cell. The cell volume was 1.42 mL. The cyclodextrin solution, with the concentrations as follows: (for FL0 C_CDs_, 1 mM; for FL2 C_αCD_, 14.2 mM; C_βCD_, 14.2 mM, C_HPβCD_, 8.52 mM), had been titrated in the cell using the automatic syringe, which also worked as a stirrer, providing homogeneous mixing of the titrated solution. The reference cell was filled with pure solvent. Each measurement consisted of 28 injections, and each injection had a volume of 10 μL. The duration of each injection was 20 s, performed at 600-s intervals, at a stirrer (syringe) speed of 351 rpm.

The thermal effects of diluting flavanones (in a cell) in DMSO (in a syringe) and the thermal effects of diluting cyclodextrins (in a syringe) with the test solvents (in a syringe) were determined independently while maintaining the same working parameters of the calorimeter, as during proper titrations of flavanone with cyclodextrin solutions. The thermal effects of the direct interaction of flavanone with cyclodextrin in a given solvent were calculated by subtracting the dilution effects of the ligand and cyclodextrin from the corresponding thermal effects of titration of the cyclodextrin with the flavanone in the same solvent.

### 3.3. Differential Scanning Calorimetry (DSC)

Measurements by differential scanning calorimetry were performed using the Chip 100 (Linseis Messgeraete GmbH, Selb, Germany) calorimeter. The test samples were placed in aluminum crucibles with a capacity of 20 µL. They were heated in the temperature range of 40 to 260 °C under a nitrogen atmosphere. The heating speed was 10 degrees/min. Measurements of pure ligand, pure cyclodextrin, and the obtained flavanone-cyclodextrin complex were performed using this method.

### 3.4. UV-Vis Spectroscopy (Phase Solubility Study)

**Figure 10:**
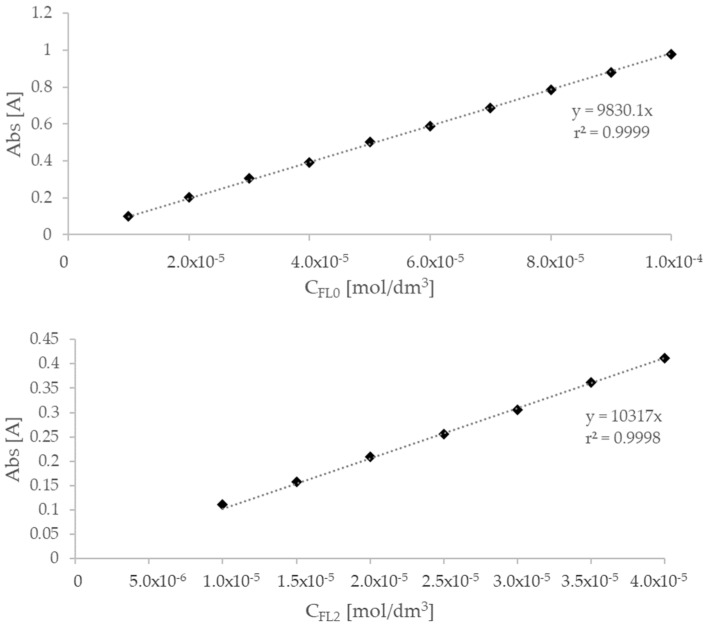
Calibration curve of flavanone and 4′-chloroflavanone for λ = 256 nm.

Spectrophotometric studies were conducted using the single-beam UV-Vis spectrophotometer SPECORD 50 (Analytik Jena, Jena, Germany). The maximum absorption of ethanolic solutions of flavanone FL0 and FL2 was marked at a wavelength of 256 nm. The calibration curve for the drugs was established for the concentration range from 1.0 × 10^−5^ M to 1.0 × 10^−4^ M. The determined molar extinction coefficient of flavanone was 9830.1 M^−1^cm^−1^ (Figure 10). The solubility in the water of this compound, established experimentally, was 12.33 mg/L (S_0_ = 5.5 × 10^−5^ mol/dm^3^). For 4′-chloroflavanone, the molar extinction coefficient was 10,317 M^−1^cm^−1^ and the solubility in pure water was 1.42 mg/L (S_0_ = 5.5 × 10^−6^ mol/dm^3^).

To investigate the effect of complex formation on the solubility of ligands in water, we have performed three measurement series. The excess ligand was placed in Eppendorf tubes containing αCD, βCD, and HPβCD solutions. The concentrations of the solutions were in the range of 1 to 90 mM for αCD, 1 to 15 mM for βCD, and 1 to 90 mM for HPβCD. The tubes were stored for 7 days at 25 °C until equilibrium. After this time, the solutions were centrifuged using a Microcentrifuge MPW-55 (MPW Med. Instruments, Warsaw, Poland) at 13,000 rpm for 5 min. Pellucid solutions were collected from above the sediment. Subsequently, they were properly diluted to ensure that the UV-Vis spectrum was within the operating range of the spectrophotometer.

### 3.5. FT-IR Spectroscopy

Spectrophotometric measurements, ranging from 450 to 4000 cm^−1^ at room temperature, were performed using a Nicolet iS5 (Thermo Fisher Scientific, Waltham, MA, USA) spectrophotometer with resolution better than 0.8 cm^−1^. The measurements were made using the transmission method, with the use of potassium bromide as a carrier for all samples. The samples, with a 200-fold excess of KBr in mass ratio, were prepared by thoroughly mixing and grinding them with a mortar. For each series, the measurements contained spectra of the pure ligand, cyclodextrin, and ligand-CD complex. The complexes were made using the co-evaporation method.

### 3.6. ^1^H NMR Spectroscopy

We performed ^1^H NMR spectroscopic analysis of the flavanones (FL0, FL2)/CD inclusion complex. Spectra of deuterated dimethylsulfoxide (DMSO-d6 purity 99.9 atom% D) with flavanones (FL0, FL2) with a concentration of 5 mM, cyclodextrins (CD) with a concentration of 5 mM, and the complex of cyclodextrin and flavanone with a concentration of 5 mM were obtained using the Avance III 600 MHz (Bruker, Billerica, MA, USA) NMR spectrometer at temperature 25 °C. All spectra were acquired under standard conditions.

## 4. Conclusions

Based on ITC measurements, it can be concluded that the inclusion complexes of both compounds: flavanone (FL0) and 4′-chloroflavanone (FL2) with all cyclodextrins (αCD, βCD, and HPβCD) were obtained in a stoichiometric ratio of 1:1. The values of the thermodynamic functions for all complexes indicate the exothermic (∆H < 0) and the spontaneous nature (∆G < 0) of the complexation processes. The complexation constant (K) determined by isothermal calorimetric titration in all the systems discussed has a value greater than 1000 (K > 1000), suggesting the formation of stable complex connections [44]. The complexation constant values determined by both methods (ITC and UV-Vis spectroscopy) show a similar trend of change, increasing in the order of αCD < HPβCD < βCD.

The constant value of the complex formation between flavanone and cyclodextrins calculated by the ITC method (Table 1 and Table 2) significantly deviates from the value determined by the solvent method of Higuchi and Connors (Table 4 and Table 5). The discrepancies in the formation constant values most probably result from the fact that the UV-Vis measurements were performed statically, while the ITC measurements were performed dynamically. The differences in the formation constant values obtained by dynamic and static methods are confirmed by previous studies [45,46].

The lower values of the constants for hydroxypropyl-β-cyclodextrin complexes, relative to the native form, may be due to the steric hindrance caused by the hydroxypropyl chains of the modified macromolecule [47,48].

UV-Vis studies showed that, for all formed complexes, a linear relationship was obtained for the increase in the concentration of flavanones as a function of the increasing concentration of the macrocycle. A similar trend can be observed in similar systems containing cyclodextrins [49,50,51]. The UV-Vis studies showed that a linear relationship of the increase in the concentration of flavanones as a function of the increase in the macrocycle was obtained for all formed complexes. This confirms the formation of complexes with a 1:1 stoichiometry.

The data we obtained under research suggest the formation of stable inclusion complexes of the tested flavanones with α-cyclodextrin, β-cyclodextrin, and hydroxypropyl-β-cyclodextrin. ITC, UV-Vis, and ^1^H NMR spectroscopy confirm and indicate the formation of the strongest interactions in the following order: βCD > HPβCD > αCD.

## Figures and Tables

**Table 1:** Values of thermodynamic parameters of FL0-CDs complexation processes at 25 °C.

	αCD	βCD	HPβCD (Mw Approximately 1380)
n	0.83 ± 0.12	0.93 ± 0.09	0.95 ± 0.10
K [dm^3^ mol^−1^]	1350 ± 130	3830 ± 490	1940 ± 190
∆H [J mol^−1^]	−179 ± 5	−109 ± 2	−120 ± 2
∆S [J mol^−1^ K^−1^]	59.5 ± 0.2	68.3 ± 0.3	62.4 ± 0.1
∆G [kJ mol^−1^]	−17.9 ± 0.1	−20.5 ± 0.2	−18.9 ± 0.2

**Table 2:** Values of the thermodynamic parameters of FL2-CDs complexation processes at 25 °C.

	αCD	βCD	HPβCD (Mw Approximately 1380)
n	0.91 ± 0.09	0.92 ± 0.12	0.98 ± 0.11
K [dm^3^ mol^−1^]	1130 ± 180	3160 ± 220	1640 ± 350
∆H [J mol^−1^]	−103 ± 2	−158 ± 5	− 150 ± 3
∆S [J mol^−1^ K^−1^]	58.0 ± 0.3	66.5 ± 0.2	61.1 ± 0.3
∆G [kJ mol^−1^]	−17.4 ± 0.2	−19.9 ± 0.2	−18.4 ± 0.1

**Table 4:** Values of the increase in the solubility of flavanone in water in the presence of selected cyclodextrins in the range of up to 15 mM and up to 90 mM, values of stability constants of the inclusion complexes, and complexation efficiency at 25 °C.

	αCD	βCD	HPβCD
Solubility increase 0–15 mM	1.6 ± 0.2	8.3 ± 0.6	17.3 ± 0.6
Solubility increase 0–90 mM	4.6 ± 0.4	-	98.7 ± 8.8
K [dm^3^ mol^−1^]	39.5 ± 5	1286.3 ± 84.9	1106.8 ± 84.5
CE	0.002	0.07	0.06

**Table 5:** Values of the increase in the solubility of 4′-chloroflavanone in water in the presence of selected cyclodextrins in the range of up to 15 mM and up to 90 mM, values of the stability constants of the inclusion complexes, and complexation efficiency at 25 °C.

	αCD	βCD	HPβCD
Solubility increase 0–15 mM	5.1 ± 1.0	17.1 ± 1.1	19.6 ± 1.0
Solubility increase 0–90 mM	27.4 ± 1.1	-	109.1 ± 6.9
K [dm^3^ mol^−1^]	299.4 ± 18.5	1252.0 ± 158.9	1185.9 ± 93.1
CE	0.0016	0.0067	0.0065

**Table 6:** Values of stability constants for complexes of cyclodextrins with flavonoids in water at 25 °C.

	αCD	βCD	HPβCD
flavanone (FL0)	39.5 ± 5.0	1286.3 ± 84.9	1106.8 ± 84.5
4′-chloroflavanone (FL2)	299.4 ± 18.5	1252.0 ± 158.9	1185.9 ± 93.1

**Table 7:** Chemical shifts (δ) and chemical shift differences (Δδ) of cyclodextrins and their complexes with flavanone (FL0) and 4′-chloroflavanone (FL2) in DMSO-d6.

^1^H Assignment	H1	H2	H3	H4	H5	H6	H 4,7,8	H9
δ_αCD_	4.806	3.393	4.476	3.284	3.779	3.595	-	-
δ_complex (αCD+FL0)_	4.806	3.393	4.480	3.286	3.779	3.596	-	-
∆δ	0.000	0.000	−0.004	−0.002	0.000	−0.001	-	-
δ_βCD_	4.839	3.359	4.427	*	3.631	3.580	-	-
δ_complex (βCD+FL0)_	4.835	3.355	4.472	*	3.637	3.575	-	-
∆δ	0.004	0.004	−0.045	*	−0.006	0.005	-	-
δ_HPβCD_	5.703	3.395	4.530	-	3.754	3.619	*	1.030
δ_complex (HPβCD+FL0)_	*	3.412	4.544	-	3.752	3.620	*	1.028
∆δ	*	−0.017	−0.014	-	0.002	−0.001	*	0.002
δ_αCD_	4.806	3.393	4.476	3.284	3.779	3.595	-	-
δ_complex (αCD+FL2)_	4.805	*	4.489	3.286	3.775	3.593	-	-
∆δ	0.001	*	−0.013	-0.002	0.004	0.002	-	-
δ_βCD_	4.839	3.359	4.427	*	3.631	3.580	-	-
δ_complex (βCD+FL2)_	4.806	3.392	4.480	*	3.643	3.595	-	-
∆δ	0.033	−0.033	−0.053	*	−0.012	−0.015	-	-
δ_HPβCD_	5.703	3.395	4.530	-	3.754	3.619	*	1.030
δ_complex (HPβCD+FL2)_	5.699	3.402	4.555	-	*	3.619	*	1.027
∆δ	0.004	−0.007	−0.025	-	*	0.000	*	0.003

∆δ = (δfreeCD − δcomplex); * Could not calculate due to overlapping of signal.

## Data Availability

The data presented in this study are available on request from the corresponding author.

## Supplementary Materials

The following supporting information can be downloaded at: https://www.mdpi.com/article/10.3390/molecules29133123/s1.

## References

[1] Merisko-Liversidge E., Liversidge G.G., Cooper E.R. (2003). Nanosizing: A formulation approach for poorly-water-soluble compounds. Eur. J. Pharm. Sci..

[2] Dizaj S.M., Vazifehasl Z., Salatin S., Adibkia K., Javadzadeh Y. (2015). Nanosizing of Drugs: Effect on Dissolution Rate. Res. Pharm. Sci..

[3] Elder D.P., Holm R., de Diego H.L. (2013). Use of Pharmaceutical Salts and Cocrystals to Address the Issue of Poor Solubility. Int. J. Pharm..

[4] Berge S.M., Bighley L.D., Monkhouse D.C. (1977). Pharmaceutical Salts. J. Pharm. Sci..

[5] Perioli L., Pagano C. (2012). Inorganic Matrices: An Answer to Low Drug Solubility Problem. Expert Opin. Drug Deliv..

[6] Patel M.S.N., Ahmed M.H., Saqib M., Shaikh S.N. (2019). Chemical Modification: A Unique Solutions to Solubility Problem. J. Drug Deliv. Ther..

[7] Fromming K.-H., Szejtli J. (1994). Cyclodextrin in Pharmacy.

[8] Rekharsky M.V., Inoue Y. (1998). Complexation Thermodynamics of Cyclodextrins. Chem. Rev..

[9] Crini G. (2014). Review: A History of Cyclodextrins. Chem. Rev..

[10] Liu L., Guo Q.X. (2002). The Driving Forces in the Inclusion Complexation of Cyclodextrins. J. Incl. Phenom. Macrocycl. Chem..

[11] Gergely V., Sebestyén G., Virág S. (1982). Toxicity Studies of Beta-Cyclodextrin. Proceedings of the First International Symposium on Cyclodextrins.

[12] Gerloczy A., Fonagy A., Keresztes P., Perlaky L., Szejtli J. (1985). Absorption, Distribution, Excretion and Metabolism of Universally Labelled 14-C-β-Cyclodextrin in Rat after per Os Administration. Arzneimittel-Forschung. Drug Res..

[13] Szente L., Puskás I., Sohajda T., Varga E., Vass P., Nagy Z.K., Farkas A., Várnai B., Béni S., Hazai E. (2021). Sulfobutylether-Beta-Cyclodextrin-Enabled Antiviral Remdesivir: Characterization of Electrospun- and Lyophilized Formulations. Carbohydr. Polym..

[14] Donati F. (2008). Sugammadex: A Cyclodextrin to Reverse Neuromuscular Blockade in Anaesthesia. Expert Opin. Pharmacother..

[15] Stepniak A., Belica-Pacha S., Rozalska S., Dlugonski J., Urbaniak P., Palecz B. (2015). Study on a Host-Guest Interaction of β-Cyclodextrin with Tebuconazole in Water. J. Mol. Liq..

[16] Navarro P., Nicolas T.S., Gabaldon J.A., Mercader-Ros M.T., Calín-Sánchez Á., Carbonell-Barrachina Á.A., Pérez-López A.J. (2011). Effects of Cyclodextrin Type on Vitamin C, Antioxidant Activity, and Sensory Attributes of a Mandarin Juice Enriched with Pomegranate and Goji Berries. J. Food Sci..

[17] Labib G.S. (2015). Novel Levocetirizine HCl Tablets with Enhanced Palatability: Synergistic Effect of Combining Taste Modifiers and Effervescence Technique. Drug Des. Dev. Ther..

[18] Tan Q., Zhang L., Zhang L., Teng Y., Zhang J. (2012). Design and Evaluation of an Economic Taste-Masked Dispersible Tablet of Pyridostigmine Bromide, a Highly Soluble Drug with an Extremely Bitter Taste. Chem. Pharm. Bull..

[19] Piao Z.Z., Lee M.K., Lee B.J. (2008). Colonic Release and Reduced Intestinal Tissue Damage of Coated Tablets Containing Naproxen Inclusion Complex. Int. J. Pharm..

[20] Cevher E., Açma A., Sinani G., Aksu B., Zloh M., Mülazımoğlu L. (2014). Bioadhesive Tablets Containing Cyclodextrin Complex of Itraconazole for the Treatment of Vaginal Candidiasis. Int. J. Biol. Macromol..

[21] Adamkiewicz L., Szeleszczuk Ł. (2023). Review of Applications of Cyclodextrins as Taste-Masking Excipients for Pharmaceutical Purposes. Molecules.

[22] Bawazeer N.A., Choudhry H., Zamzami M.A., Abdulaal W.H., Middleton B., Moselhy S.S. (2016). Role of Hesperetin in LDL-Receptor Expression in Hepatoma HepG2 Cells. BMC Complement. Altern. Med..

[23] Choi E.J., Lee J.I., Kim G.H. (2010). Anti-Carcinogenic Effect of a New Analogue 4′-Chloroflavanone from Flavanone in Human Breast Cancer Cells. Int. J. Mol. Med..

[24] Safavi M., Esmati N., Ardestani S.K., Emami S., Ajdari S., Davoodi J., Shafiee A., Foroumadi A. (2012). Halogenated Flavanones as Potential Apoptosis-Inducing Agents: Synthesis and Biological Activity Evaluation. Eur. J. Med. Chem..

[25] (2007). Dimethyl Sulfoxide (DMSO) Health and Safety Information.

[26] Otterbach A., Lamprecht A. (2021). Enhanced Skin Permeation of Estradiol by Dimethyl Sulfoxide Containing Transdermal Patches. Pharmaceutics.

[27] Bragger J.M., Dunn R.V., Daniel R.M. (2000). Enzyme activity down to -100 degrees C. Biochim. Biophys. Acta.

[28] Buvári-Barcza A., Barcza L. (1999). Influence of the guests, the type and degree of substitution on inclusion complex formation of substituted betacyclodextrins. Talanta.

[29] Jambhekar S.S., Breen P. (2016). Cyclodextrins in pharmaceutical formulations II: Solubilization, binding constant, and complexation efficiency. Drug Discov. Today.

[30] Koester L.S., Guterres S.S., Le Roch M., Eifler-Lima V.L., Zuanazzi J.A., Bassani V.L. (2021). Ofloxacin/β-Cyclodextrin Complexation. Drug Dev. Ind. Pharm..

[31] Wen J., Liu B., Yuan E., Ma Y., Zhu Y. (2010). Preparation and Physicochemical Properties of the Complex of Naringenin with Hydroxypropyl-β-Cyclodextrin. Molecules.

[32] Rao V.K., Rao M.S., Kumar A. (2011). Ytterbium(III) Triflate: An Efficient and Simple Catalyst for Isomerization of 2′-Hydroxychalcone and 2′-Aminochalcones in Ionic Liquid. J. Heterocycl. Chem..

[33] Kostrzewa-Susłow E., Dmochowska-Gładysz J., Białońska A., Ciunik Z. (2008). Microbial Transformations of Flavanone by Aspergillus Niger and Penicillium Chermesinum Cultures. J. Mol. Catal. B Enzym..

[34] Zheng X., Jiang H., Xie J., Yin Z., Zhang H. (2013). Highly Efficient and Green Synthesis of Flavanones and Tetrahydroquinolones. Synth. Commun..

[35] Higuchi T., Connors K.A. (1965). Phase Solubility Techniques. Adv. Anal. Chem. Instrum..

[36] Tao Y., Han Y., Dong S., Fan X., Wang T., Yan X. (2018). Preparation, Solubilization and In vitro Anti-tumour Effect of Water-soluble Betulinic Acid/Oligo(polylvinylamino) Bridged bis(β-cyclodextrin)s Complexes. Chem. Sci. Int. J..

[37] Cui L., Zhang Z.H., Sun E. (2012). Effect of β-cyclodextrin complexation on solubility and enzymatic conversion of naringin. Int. J. Mol. Sci..

[38] Jung H., Shin S.Y., Jung Y., Tran T.A., Lee H.O., Jung K.Y., Koh D., Cho S.K., Lim Y. (2015). Quantitative Relationships between the Cytotoxicity of Flavonoids on the Human Breast Cancer Stem-Like Cells MCF7-SC and Their Structural Properties. Chem. Biol. Drug Des..

[39] Kim J.S. (2020). Study of Flavonoid/Hydroxypropyl-β-Cyclodextrin Inclusion Complexes by UV-Vis, FT-IR, DSC, and X-ray Diffraction Analysis. Prev. Nutr. Food Sci..

[40] Qiu N., Cheng X., Wang G., Wang W., Wen J., Zhang Y. (2014). Inclusion complex of barbigerone with hydroxypropyl-β-cyclodextrin: Preparation and in vitro evaluation. Carbohydr. Polym..

[41] Thakkar A.L., Demarco P.V. (1971). Cycloheptaamylose inclusion complexes of barbiturates: Correlation between proton magnetic resonance and solubility studies. J. Pharm. Sci..

[42] Greatbanks D., Pickford R. (1987). Cyclodextrins as chiral complexing agents in water, and their application to optical purity measurements. Magn. Reson. Chem..

[43] Silva I.S., Feitosa E.L., Santos M.E.P., Silva R.M., Rocha M.S., da Silva F.I., Lima F.C.A., Costa A.M.S., Alves P.B., de Sousa S.A.A. (2020). Theoretical and Experimental Investigations on Inclusion Complex β-Cyclodextrin and Sulcatone: A Cardiovascular Activity Evaluation. J. Braz. Chem. Soc..

[44] Biernacka M., Ilyich T., Zavodnik I., Palecz B., Stepniak A. (2022). Studies of the Formation and Stability of Ezetimibe-Cyclodextrin Inclusion Complexes. Int. J. Mol. Sci..

[45] Buczkowski A., Urbaniak P., Palecz B. (2012). Thermochemical and spectroscopic studies on the supramolecular complex of PAMAM-NH2 G4 dendrimer and 5-fluorouracil in aqueous solution. Int. J. Pharm..

[46] Buczkowski A., Urbaniak P., Piekarski H., Palecz B. (2017). Spectroscopic and calorimetric studies on the interaction between PAMAM G4-OH and 5-fluorouracil in aqueous solution. Spectrochim. Acta Part A Mol. Biomol. Spectrosc..

[47] Stella V.J., Rajewski R.A. (1997). Cyclodextrins: Their future in drug formulation and delivery. Pharm. Res..

[48] Fourmentin S., Ciobanu A., Landy D., Wenz G. (2013). Space filling of β-cyclodextrin and β-cyclodextrin derivatives by volatile hydrophobic guests. Beilstein J. Org. Chem..

[49] Dhruve P., Tripathi A., Gidwani B., Vyas A. (2017). Investigating the phase complexed with -solubility and compatibility study of Anticancer drug β-cyclodextrin and hp–β-cyclodextrin. Int. J. Adv. Pharm. Sci..

[50] Saokham P., Muankaew C., Jansook P., Loftsson T. (2018). Solubility of Cyclodextrins and Drug/Cyclodextrin Complexes. Molecules.

[51] Soe H.M.S.H., Kerdpol K., Rungrotmongkol T., Pruksakorn P., Autthateinchai R., Wet-osot S., Loftsson T., Jansook P. (2023). Voriconazole Eye Drops: Enhanced Solubility and Stability through Ternary Voriconazole/Sulfobutyl Ether β-Cyclodextrin/Polyvinyl Alcohol Complexes. Int. J. Mol. Sci..

