# Supplementary material for: Host-Guest Complexes of Flavanone and 4′-Chloroflavanone with Naturals and Modified Cyclodextrin: A Calorimetric and Spectroscopy Investigations
Source: Molecules. 2024 Jun 30;29(13):3123. doi: 10.3390/molecules29133123 (PMC11243463; doi:10.3390/molecules29133123)
Supplement: Supplementary file 1 [file molecules-29-03123-s001.zip › molecules-3055297-Supplementary material.pdf]

molecules

MDPI

**Supplementary material:**

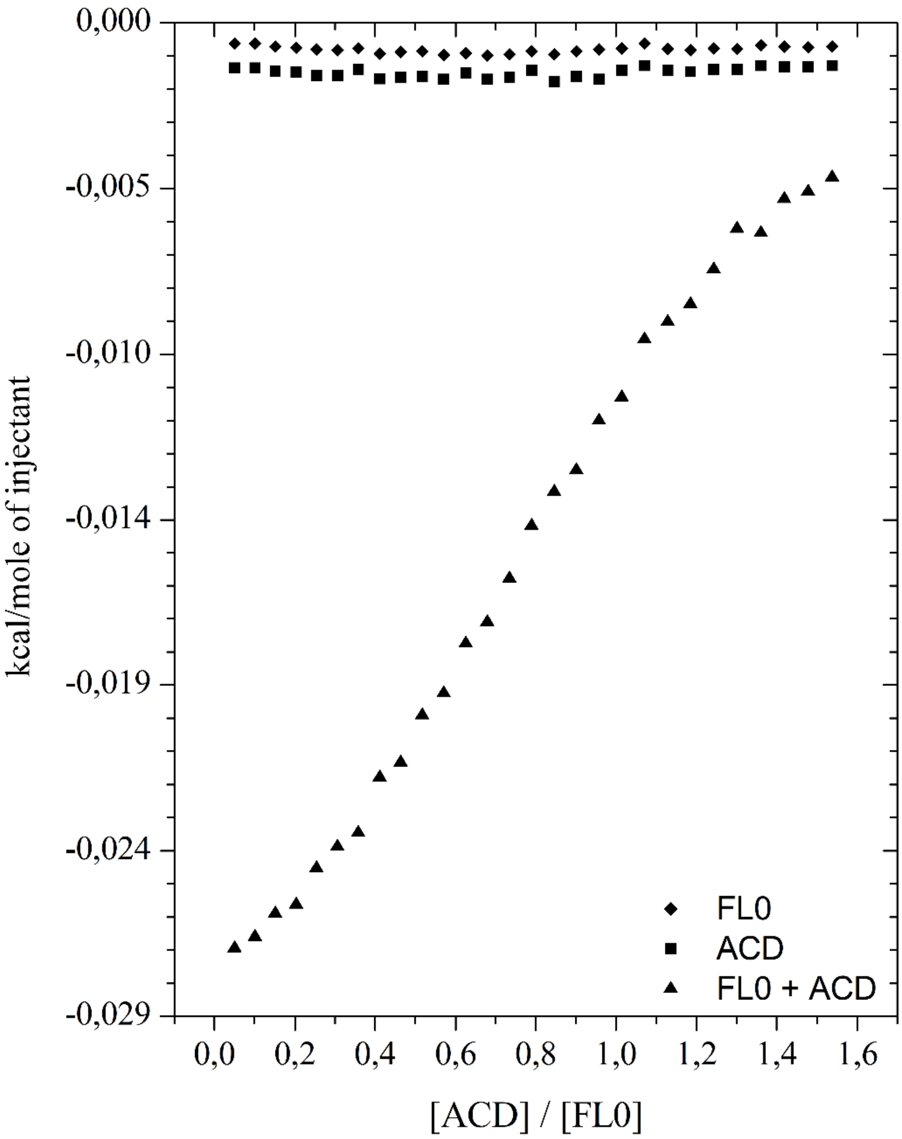


**Figure S1.** Thermal effects: ■ dilution of $\alpha$-cyclodextrin solution in DMSO; ◆ diluting a solution of flavanone (FL0) in DMSO; ▲ titration of the flavanone (FL0) solution with a solution of $\alpha$-cyclodextrin in DMSO

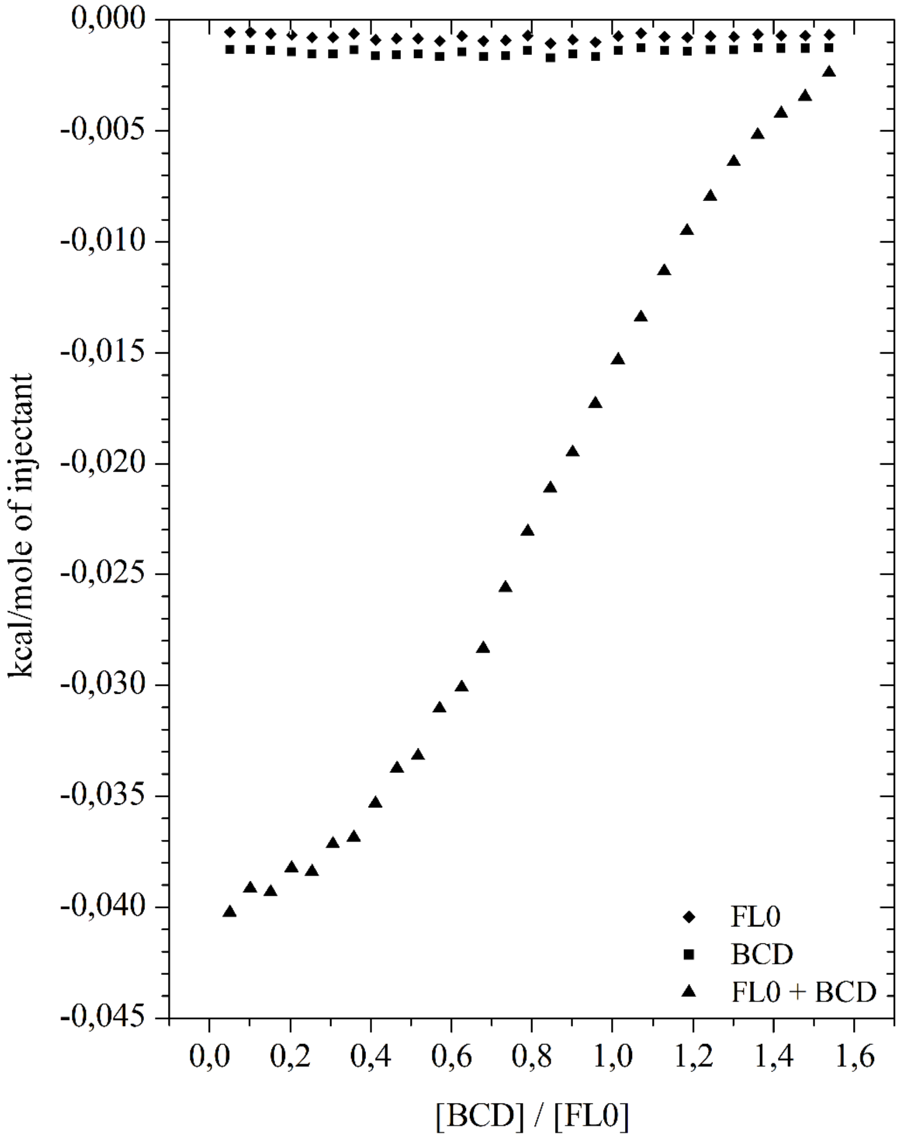


**Figure S2.** Thermal effects: ■ dilution of β-cyclodextrin solution in DMSO;◆ diluting a solution of flavanone (FL0) in DMSO; ▲ titration of the flavanone (FL0) solution with a solution of β-cyclodextrin in DMSO

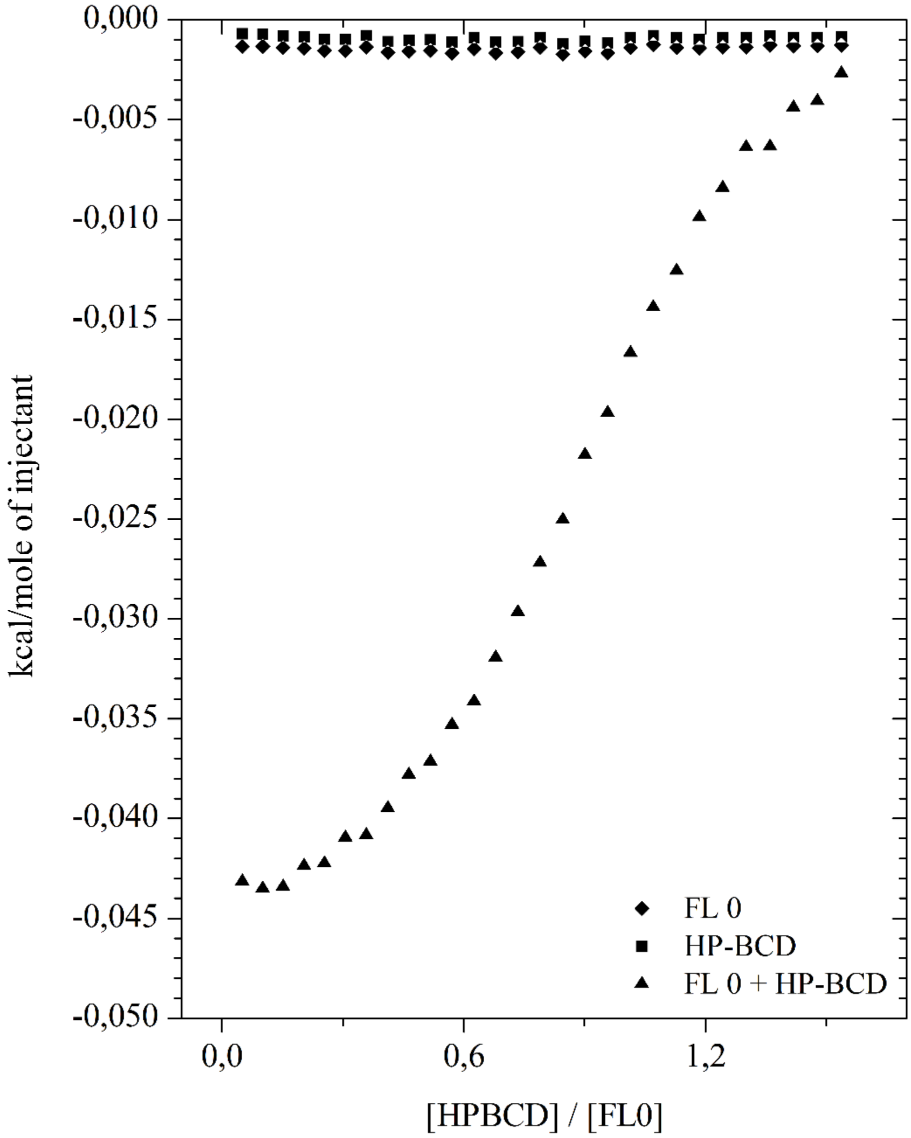


**Figure S3**. Thermal effects: ■ dilution of HP-β-cyclodextrin solution in DMSO;◆ diluting a solution of flavanone (FL0) in DMSO; ▲ titration of the flavanone (FL0) solution with a solution of HP-β-cyclodextrin in DMSO

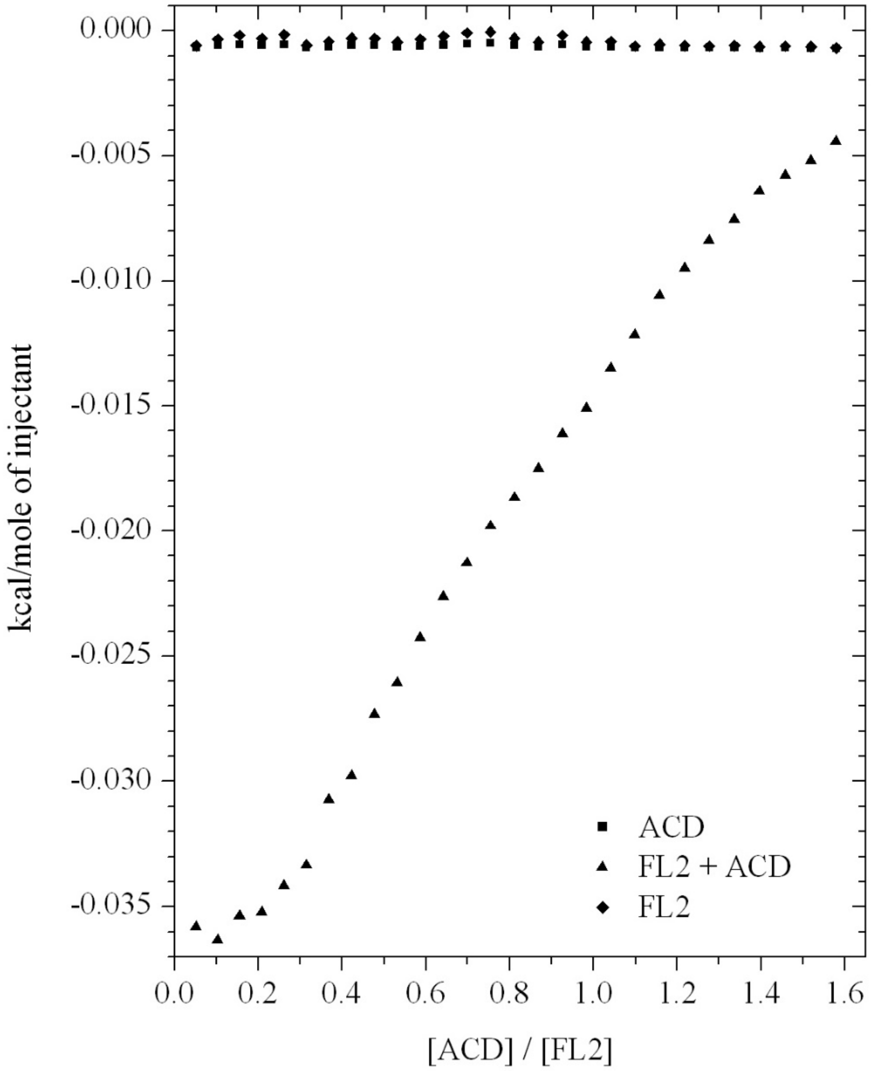


**Figure S4.** Thermal effects: ■ dilution of $\alpha$-cyclodextrin solution in DMSO;◆ diluting a solution of flavanone (FL2) in DMSO; ▲ titration of the flavanone (FL2) solution with a solution of $\alpha$-cyclodextrin in DMSO

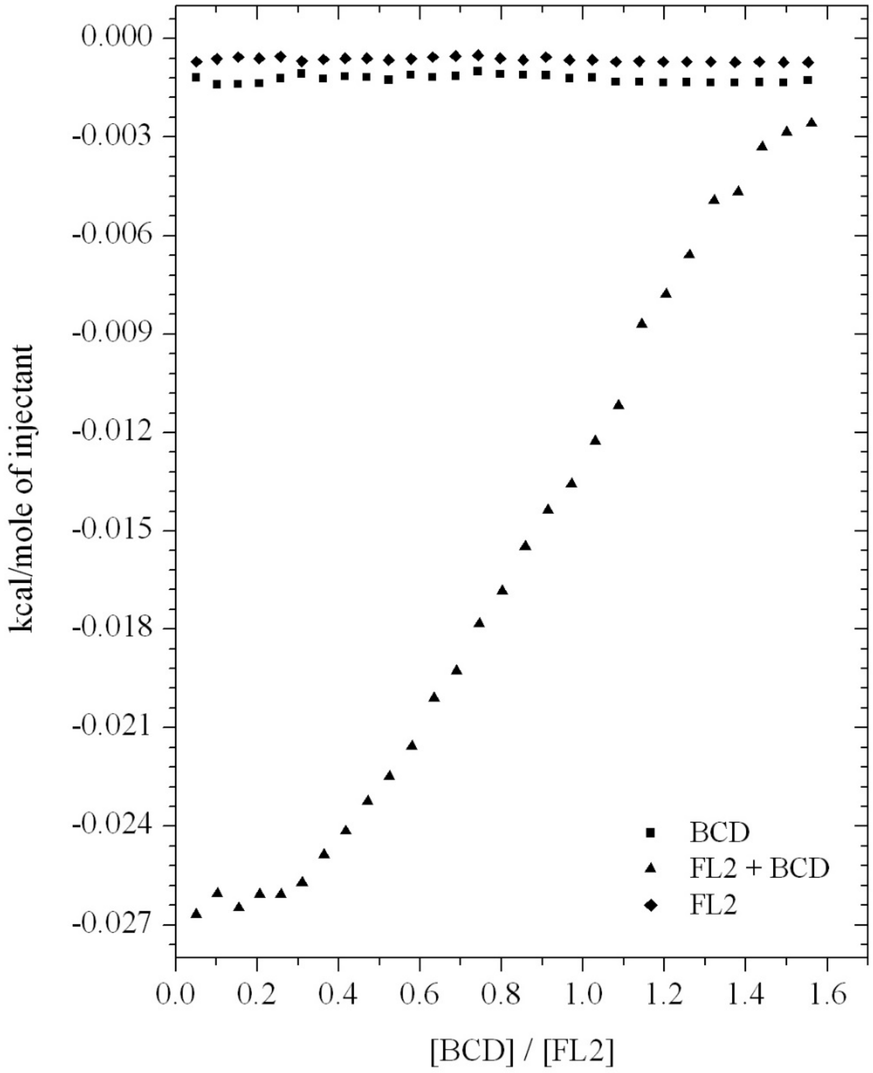


**Figure S5.** Thermal effects: ■ dilution of β-cyclodextrin solution in DMSO; ◆ diluting a solution of flavanone (FL2) in DMSO; ▲ titration of the flavanone (FL2) solution with a solution of β-cyclodextrin in DMSO

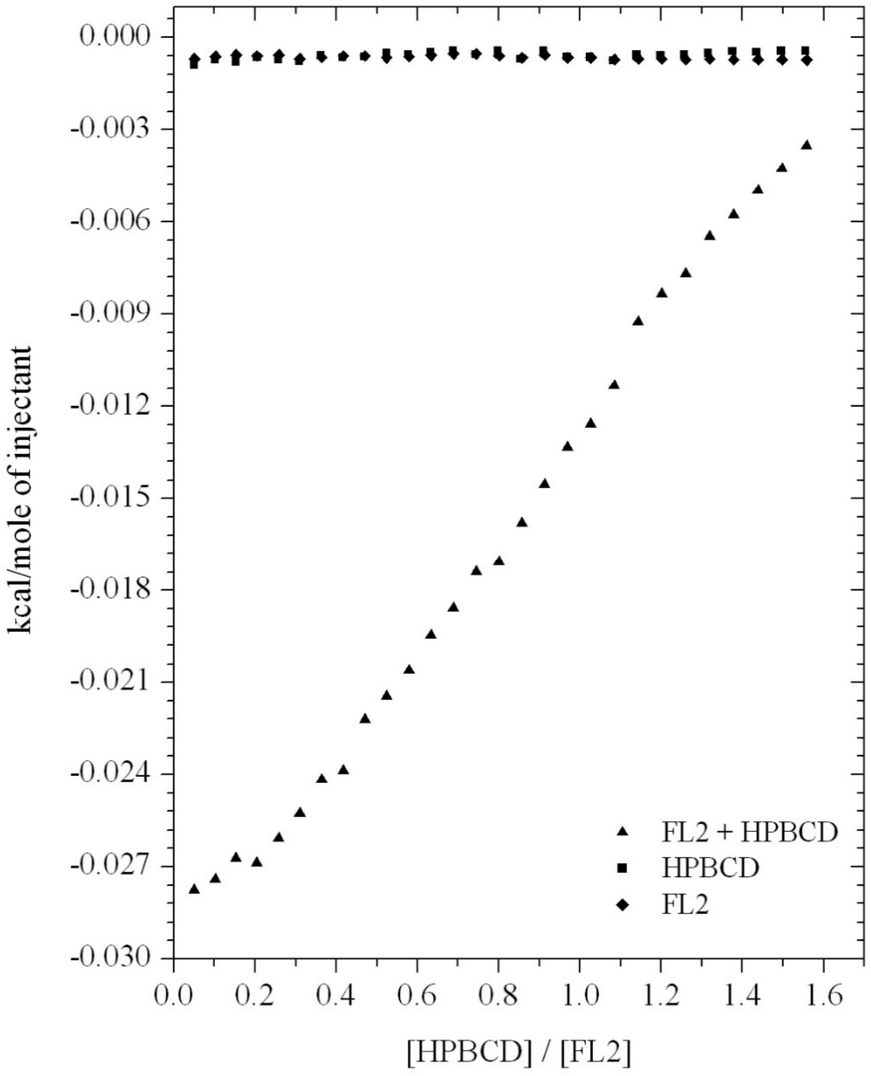


**Figure S6.** Thermal effects: ■ dilution of HP-β-cyclodextrin solution in DMSO; ◆ diluting a solution of flavanone (FL2) in DMSO; ▲ titration of the flavanone (FL2) solution with a solution of HP-β-cyclodextrin in DMSO

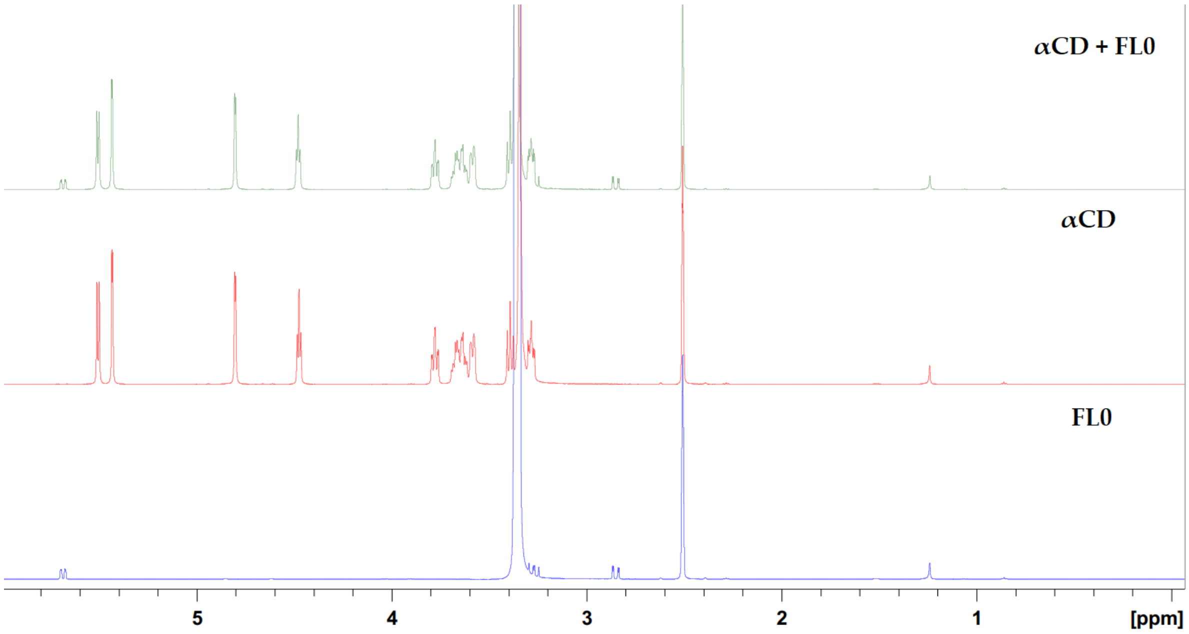


**Figure S7.** $^{1}$HNMR spectra of : α-CD/FL0 complex; α-CD; FL0

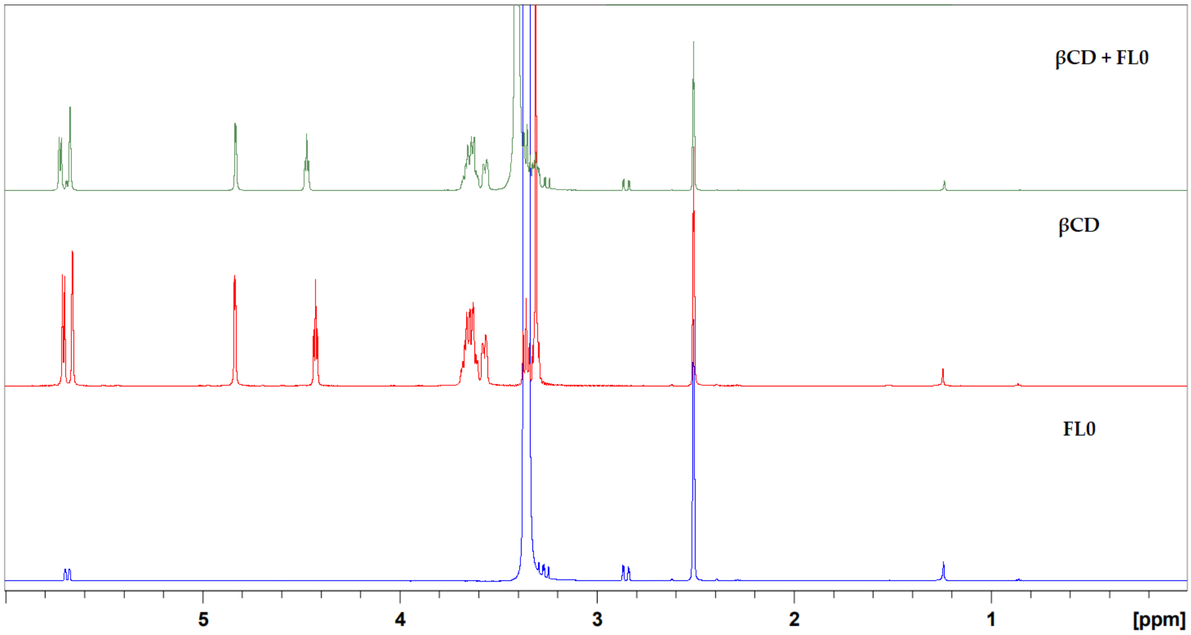


**Figure S8.** $^{1}$HNMR spectra of : β-CD/FL0 complex; β-CD; FL0

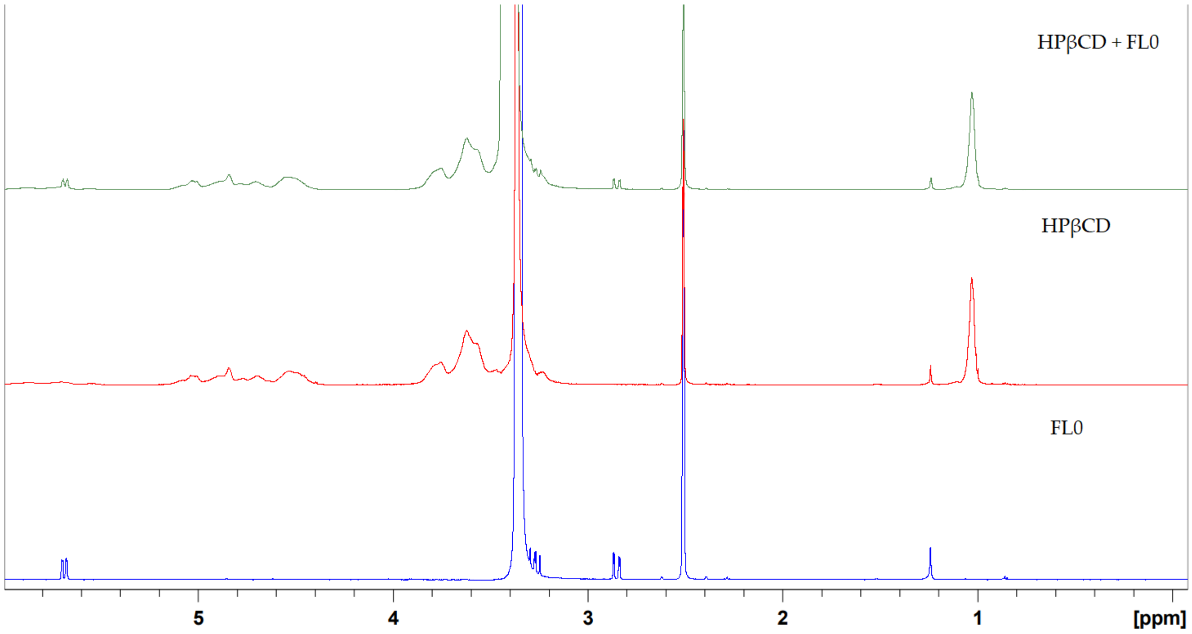


**Figure S9.** $^{1}$HNMR spectra of : HPβ-CD/FL0 complex; HPβ-CD; FL0

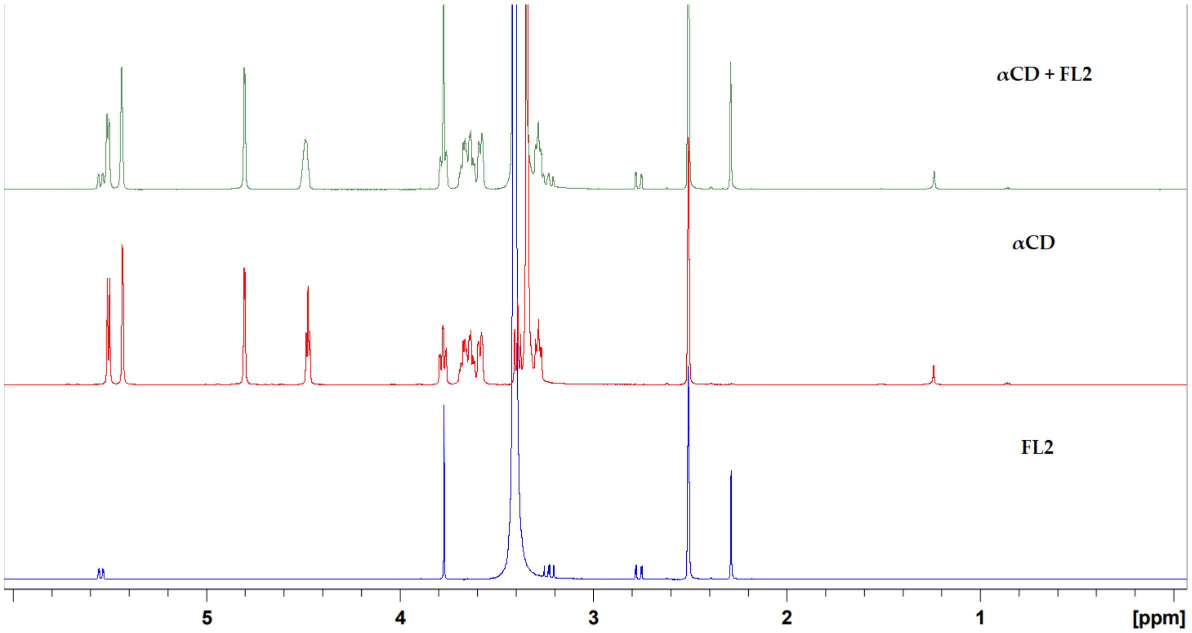


**Figure S10.** $^{1}$HNMR spectra of : α-CD/FL2 complex; α-CD; FL2

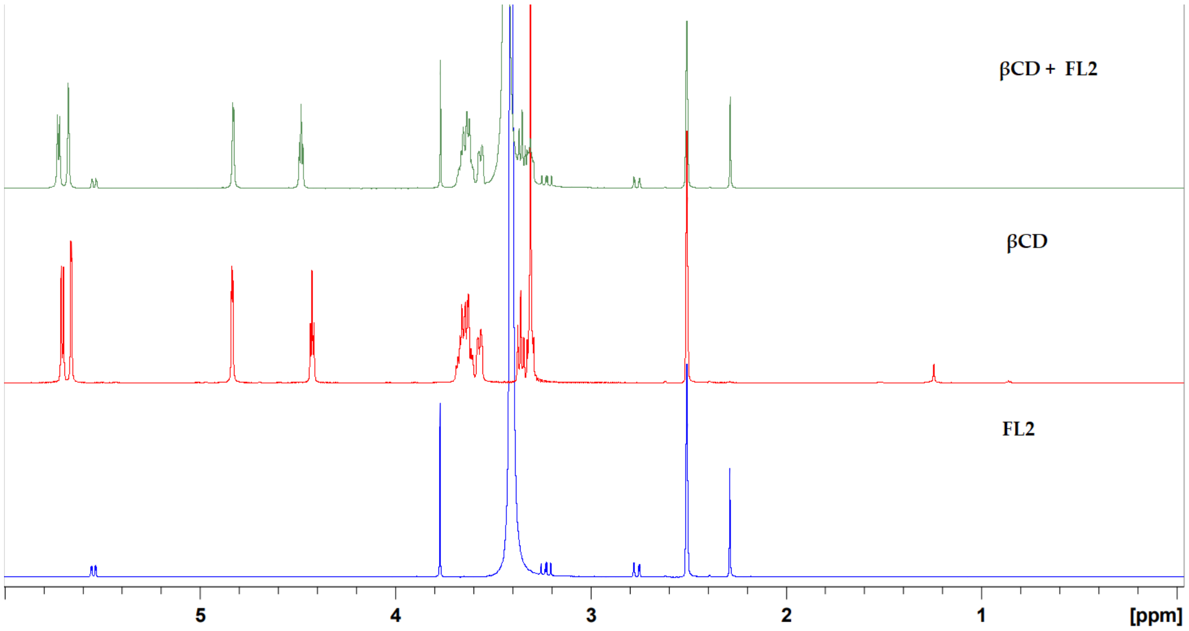


**Figure S11.** $^{1}$HNMR spectra of : β-CD/FL2 complex; β-CD; FL2

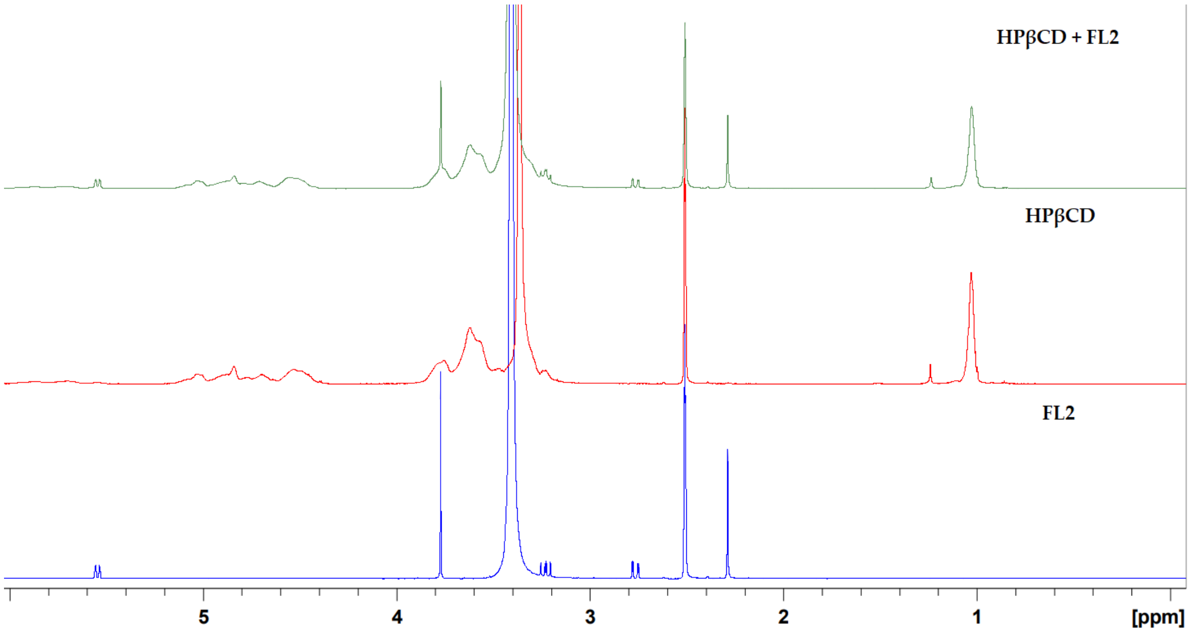


**Figure S12.** $^{1}$HNMR spectra of : HPβ-CD/FL2 complex; HPβ-CD; FL2

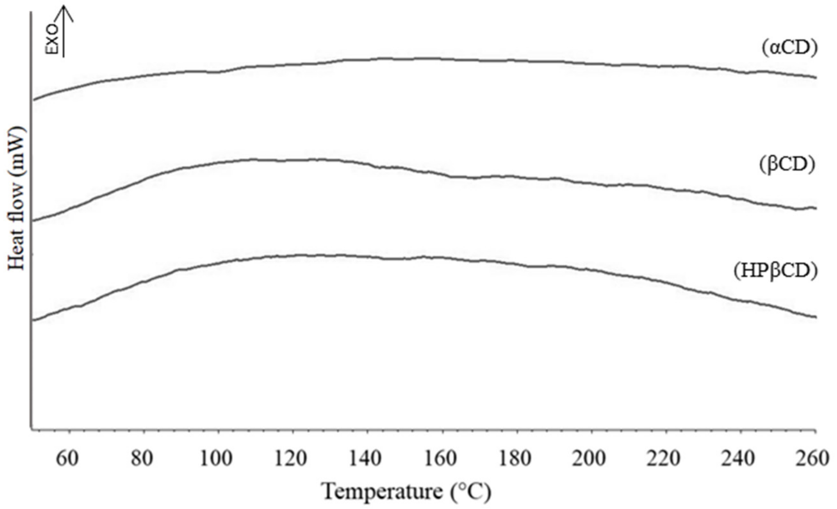


**Figure S13** Thermograms of pure cyclodextrins.

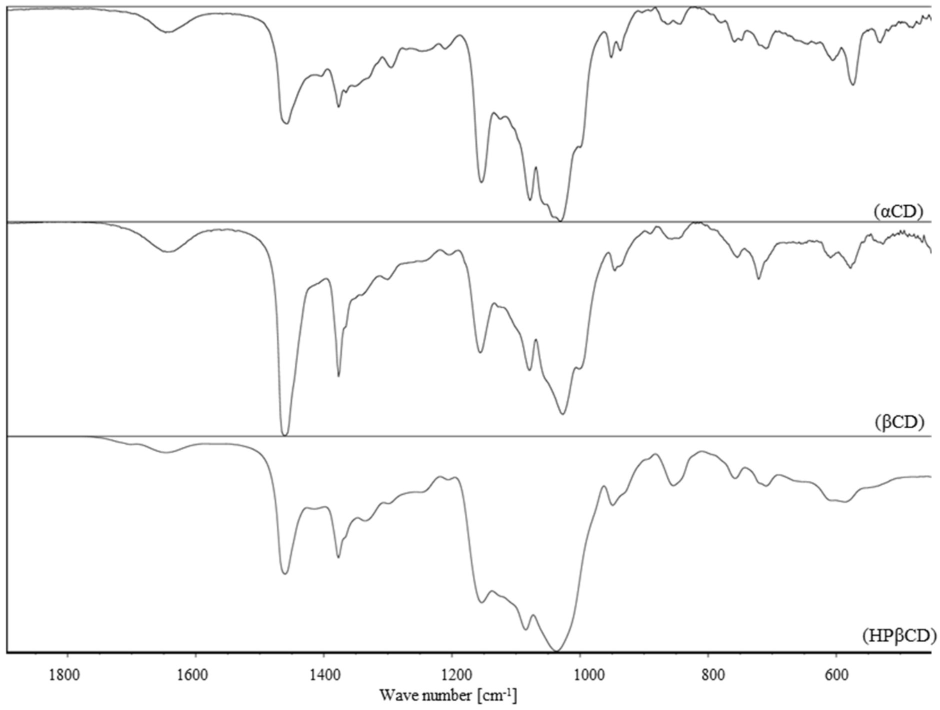


**Figure S14** The FTIR spectrum of pure cyclodextrins.
